# “It still haunts me whether we did the right thing”: a qualitative analysis of free text survey data on the bereavement experiences and support needs of family caregivers

**DOI:** 10.1186/s12904-016-0165-9

**Published:** 2016-11-08

**Authors:** Emily Harrop, Fiona Morgan, Anthony Byrne, Annmarie Nelson

**Affiliations:** 1 Division of Population Medicine, Marie Curie Palliative Care Research Centre, Cardiff University School of Medicine, 1st Floor Neuadd Meirionydd, Heath Park Way, Cardiff, CF14 4YS UK; 2 SURE/ School of Healthcare Sciences, Cardiff University, 1st Floor Neuadd Meirionydd, Heath Park Way, Cardiff, CF14 4YS UK

**Keywords:** Palliative care, End of life care, Caregivers, Bereavement, Grief, Qualitative

## Abstract

**Background:**

Research suggests that there may be bereavement experiences and support needs which are specific to family caregivers providing end of life care (EoLC), although this remains an under-researched area. This paper focuses on themes relating to bereavement which were derived from an analysis of free text survey responses collected in a research priority setting exercise for palliative and EoLC.

**Methods:**

The priority setting exercise involved a public survey, designed to generate research priorities. Rather than identify research topics, many people instead described their experiences and raised more general questions relating to palliative and end of life care. To explore these experiences and perspectives a supplementary thematic analysis was conducted on the survey responses. 1403 respondents took part, including patients, current and bereaved carers, health and social care professionals, volunteers and members of the public.

**Results:**

Several grief issues were identified, which seem specific to the experiences of family caregivers. Responses demonstrated a relationship between death experiences, feelings of guilt and bereavement outcomes for some family caregivers, as well as caregiver experiences of a “void” created by the withdrawal of professional support after death. Communication and support needs were also identified by participants.

**Conclusion:**

This analysis provides further evidence of some of the specific effects that caring for a loved one at the end of life can have on bereavement experiences. Finding ways of improving communication around the time of death and effective follow up approaches post death could help to address some of these issues.

## Background

Palliative care is recognised to have an important role to play in addressing the support needs of bereaved families [1–5]. Although grief is a natural process, in which most people learn to adjust, around nine percent of adults experiencing a loss develop complicated grief reactions [6]. These reactions have been described as painful and persistent responses associated with mental and physical health problems [6], and for bereaved caregivers, estimations of the proportion of the population who experience complicated grief range from between 10 and 20 % [1]. However, the relationship between caregiving and bereavement remains under-researched [7, 8], and the process of adaptation to loss often decontextualized, without consideration of the experience of caregiving or of the death [8].

A number of quantitative studies have demonstrated relationships between caregiving experiences and long term health and quality of life outcomes for bereaved caregivers [9–13], while the available qualitative evidence also illustrates some of the ways in which caregiving impacts upon bereavement. Although caregivers in some studies described positive outcomes such as feelings of privilege, accomplishment, expressions of love and improved family relationships [8, 14–19], these and other study findings have also pointed to caregiver burden and the physical and emotional exhaustion experienced by carers at the time of death [7, 14, 20, 21]. Former caregivers have also reported feelings of failure, guilt and regret in relation to unfulfilled place of care preferences and decision-making responsibilities and outcomes [15, 19, 20, 22, 23]. These accompany haunting images of physical and emotional suffering associated with the illness [8, 12, 24, 25], the physical and cognitive decline of the patient [8, 15, 25] and the trauma of the death itself [17, 25]. Other factors that have been found to impact upon bereavement and complicate the grieving process for family caregivers include missing the death, lack of preparedness for death [12, 20, 25], inadequate terminal support [20, 24, 25] and insufficient knowledge of patient history amongst treating healthcare professionals [20].

This paper reports on a supplementary qualitative analysis of ‘free text’ (open ended) survey data relating to bereavement, which was collected from a recent research priority setting exercise for palliative and end of life care (EoLC) research [26]. The results reported in this paper provide further insight into how carer specific experiences of EoLC and the death of their loved ones impact upon their bereavement experiences. Support needs and clinical implications are also identified.

## Methods

In 2014 the Palliative and end of life care Priority Setting Partnership (PeolcPSP) carried out a public survey of 1403 people with the aim of identifying unanswered questions in palliative and EoLC (see Table 1 for breakdown of respondents). Using an electronic questionnaire made available on Survey Monkey and a paper version made available in hospices and via the Marie Curie Nursing Service, respondents were invited to ask questions about palliative and EoLC, as displayed in Table 2. A purposeful approach to sampling was used; the survey was disseminated to stakeholder networks via email, newsletters, social media, web posts, presentations and stands at conferences, and blogs, as described elsewhere [26]. The process followed a method developed by the James Lind Alliance for setting research priorities, which has typically been used to identify research questions concerned with evaluating clinical interventions [27]. Following this approach and completion of the exercise a ‘top ten’ list of research priorities for Palliative and EoLC in the UK was produced, and is available in an on-line report [26].

**Table 1:** Numbers of different groups responding to the PeolcPSP survey; multiple answers were possible

Respondent (Reporting ID)	Survey responses (*n* = 1403)	Responses relating to bereavement (*n* = 154)
I am in the last few years of my life (Patient)	59	6
I am a carer or family member or partner or friend of someone in the last few years of their life (Current carer)	176	20
I am a bereaved carer or family member or friend (Bereaved Carer)	494	76
I am a professional working with people in the last few years of life (Professional)	680	68
I am a volunteer working with people in the last few years of life (Volunteer)	43	3
I am a member of the public who has an interest in the subject (Member of Public)	181	11
Other	142	4

**Table 2:** Survey questions on palliative and EoLC

Q. What questions do you have about care, support and treatment of people who are in the last few years of their lives that could help them to live as well as possible? This could also include question(s) about care and support for current carers or families.	
Q. What questions do you have about care, support and treatment of people for those rapidly approaching the end of their lives? This could also include question(s) about care and support for current or bereaved carers or families looking after someone at the end of life.	

However, it became clear whilst analysing the survey responses that not all responses could be translated into questions that might be answered by intervention based studies. Many people described their experiences and raised more general questions, for example about the purpose of palliative care. The PeolcPSP Steering Group felt strongly that these responses (which were considered ‘out of scope’ of the original survey analysis) should not be lost and that the voices of everyone who took part in the survey be heard. In particular, it was felt that the detailed, experiential nature of many of the ‘free text’ responses to the survey questions could provide valuable insight into many topics of interest in current palliative and end of life care research, if analysed and reported using a thematic approach.

Once the prioritisation exercise was completed all survey responses were subjected to a supplementary thematic analysis. The initial coding framework was developed using an inductive approach, after two researchers reviewed 200 printed copies of the survey responses. The coding framework was applied to 50 survey responses to confirm that it reflected the data. Researchers entered the coding framework into NVivo 10 software and systematically coded the survey responses into broad thematic areas using established techniques of coding and comparison [28]. More descriptive codes were then created and applied to the ‘bereavement’ data set by the author (EH) following the same techniques, and were checked by another member of the research team (AN).

## Results

### Death and EoLC experiences and impacts on bereavement

Many participants described how the traumatic deaths of their loved ones directly affected their bereavement experiences, preventing them from being able to grieve ‘properly’, and leaving unanswered questions and enduring feelings of guilt and regret. Participants gave powerful descriptions of perceived physical suffering in their family members, including one patient “fighting against” and “awaking frightened” of the syringe driver and another “gasping for breath”. There were also multiple references to the Liverpool Care Pathway (LCP). This care pathway was developed and implemented in the UK to improve the quality of care provided to patients in hospitals in the final days or hours of life [29], but was withdrawn in 2013 following controversy surrounding its implementation [30]. A number of bereaved family members, whose relatives had been placed on the Pathway, described their concerns over the perceived “denial” of food and water, the fact that patients were unable to communicate their needs and uncertainty over whether they were suffering as a result of this. Respondents were also concerned over their lack of understanding of, or inclusion in, the dying process and described feeling “haunted” by memories of the death. They were left with nagging doubts over whether the right decisions were made and whether they could have done more for their loved ones, including in some cases to fulfil patient wishes for a home death. For the family below, the recent withdrawal of the LCP added to these doubts by giving confirmation to their suspicions of the pathway:

*My mother died of breast cancer in the hospice… My questions would have been about the Liverpool pathway - it still haunts me whether we did the right thing, and now that it has been stopped, I live with a terrible feeling of guilt that my suspicions were right…. My mother kept trying to speak to me but was too weak, and I couldn't make out what she was saying. I am so afraid that she was asking for water. … my mother wanted (to die at home) and I would love to have been able to fulfill that wish. I live with that regret.* (Current Carer and Bereaved Carer)

*We as a family have not been able to grieve for our mother who was taken away from us she was put to death on the LCP and nothing was explained, we were told this is whats going to happen now!! There was no dignity watching my mother gasp for breath over 4 days, she was denied food and water why was this?* (Current Carer and Bereaved Carer)

Several respondents also gave examples of what they felt to be good EoLC and death experiences in the context of dealing with bereavement. These included hospice and home deaths, with effective multi-professional support in the weeks preceding the death, and follow up contact and offers of support post-death. This support enabled family members to feel certain that their loved ones were receiving the best possible care and also helped alleviate their own tiredness. Respondents directly related these experiences to their own emotional recovery and adjustment. This was contrasted with the difficult death and grief experiences of other friends and family members:

*I have experienced the loss of two members of my family one person died at home with the support of our GP, District Nurses, carers and Marie Curie nurses. I recovered emotionally from this experience quicker as I felt my mother had the best of care and support and I was not as not as physically exhausted as when my sister-in-law died. My sister-in-law died in hospital, she was put on the Liverpool Care Plan - this was not explained fully to my brother-in-law and the whole experience has left him emotionally fragile and requiring our support.* (Bereaved Carer)

*My wife (aged 79 then) died rather suddenly of a very aggressive cancer of the uterus… In the final weeks we both fear that the care that one received could not have been better. I am thus prompted to reply to you saying ‘what went right’ ….. Since (wife’s) death the hospice has sent me repeated message offering bereavement counselling - which I did not feel I needed. On the day following her death a small team met me for 1 1/2 hours to give me death certificate and also the various things I had to do….So many of our peers have very different experiences!!* (Bereaved Carer and Member of the Public)

### Improved communication during the dying stages and post death

The bereavement issues caused by poor communication and lack of understanding or awareness of the dying process and EoLC are evident. It follows that a number of suggestions were made by caregivers and health professionals for improving communication and support for families at the end of life. One of these was for improved information and communication on the dying process. Carers expressed wishes for bedside updates on what was happening and what they could be doing to help their loved ones at that time. Carers and health professionals also described needs for better information on the signs of death and what to expect in order to facilitate their preparedness for the death. This need was emphasised for families of patients with long-term conditions such as Alzheimer’s, and seemed to be particularly acute for the wife of a long term Multiple Sclerosis (MS) patient. She had become so accustomed to caring for her heavily disabled husband that his death came as a shock, for which she was totally unprepared:

*I would have liked more guidance in the final moments of my loved one’s life. Perhaps someone with me at the bedside from time to time to let me know what was happening and what I could do. I think this is most important for a carer or family who has not experienced the death of a loved one before.* (Bereaved Carer)

*My husband had MS for over 30 years. For the final 13 he was quadriplegic and unable to speak. A year before he died he lost the ability to swallow. He died quite suddenly after 8 days of altered breathing. I did not realise he was actually dying until the day previous to his death. He died at home with me being his sole carer throughout…. I wish now that somebody had sat down and talked to me about what was happening. It wouldn't have changed the outcome but I was totally unprepared for his death. I had been caring for so long but had never talked to anybody about the end of his life despite his deteriorating condition.* (Bereaved Carer)

Carers and health professionals also described their needs for more effective communication and support around EoLC planning and decisions. These included better explanation of what interventions such as the LCP involve so that families have clearer understandings of what will happen to their loved ones once the focus of care has shifted to prioritising EoLC needs, as well emotional support to help them come to terms with these decisions:

*How carers can cope with decisions by their loved one to hasten end of life by refusing food, drink or medications that would almost certainly prolong life.* (Bereaved Carer; work for a charity supporting people with a life limiting condition)

*The palliative care services we experienced were first rate on the whole, however I think families of dying patients would benefit from research on ways to support them in coming to terms with the withdrawal of IV drips and hydration in the last days of life. I’m convinced this is the source of much dissatisfaction with end of life care.* (Bereaved Carer)

The need to more effectively engage families in discussions around the death so that their concerns are addressed at the time, subsequently reducing impact on the grieving process, was also raised by a health professional/family carer:

*Are families happy with TLC interventions and removal of medical intervention and monitoring? Everyone is different but what is the best way to broach this with relatives at this emotive time? How can we get families to express their wishes, concerns so they can be dealt with effectively? Unexpressed niggling concerns could affect the grief process that could have been very easily dealt with at the time (I understand some will not be present at the time).* (Current Carer and Professional)

Some respondents took this one step further and called for post death consultations so that families’ questions could be answered by the health professionals who were directly involved at the end of life. It was felt that this would alleviate some of the confusion and concerns of bereaved family members and provide an opportunity to improve future care:

*After a death could it be possible to talk to a doctor about what happened at the end and explain what was happening as the bodies functioning breaks down?* (Bereaved Carer)

*Value should be given to the possibility of nurses who were involved in the care of the dead patient to make one or two visits to allow the bereaved to talk over any confusions/issues in how it all went.* (Bereaved Carer and Professional)

### Living in a “void”: the need for continuity of care

Many carers highlighted the need for greater continuity of care post-death, from the services and people with whom they had built relationships during the end of life period. Respondents reflected on feeling “cut off” and on the “void” that is left following the withdrawal of support. This occurs at a time when they are already struggling to come to terms with the loss of their loved ones, and in some cases the caring role and identity that has defined their lives for so long:

*Sadly when someone dies, not only is there a huge hole where their loved one was, but also an immediate void from all the HSCP’s involved, understandably, but maybe there needs to be mechanism whereby they are not left to there own devices (unless they want to of course) sometimes creating mental health issues, which is not helpful to anyone.* (Bereaved Carer and Volunteer)

*It was a huge shock after caring for my husband for 24 hours a day for 20 years to lose him. Not only did I lose my husband but also my whole purpose to live myself. He was also my “full time job”. I felt totally lost when I lost him. We had regular contact with various professionals when he was alive ie DNs, the hospice, dietitians, stoma nurse, speech therapist, GPs (very regularly). Suddenly, when my husband died, all this stopped. My income also stopped the day he died as he was in receipt of disability benefits. I felt totally lost and abandoned.* (Bereaved Carer)

A number of health professionals also felt that they should be providing more support to family members post death, similarly reflecting on the negative impact of withdrawal of services and the need to have continued support available for those that need it:

*I often think that as a Health Care Professional that we do not follow up post the death of our patients’re their families, and loved ones more so if we have been present in the final hours. How do we not ??* (Professional)

*….they (relatives) are left with the emptiness that prevails following a stream of differing care professionals having been in their home occasionally for many months at a time just stopping overnight, this can be very depressing for many people young and old. (Professional)*

A large number of caregivers and professional respondents also asked questions about the availability of bereavement support and described difficulties accessing support, suggesting a lack of information and/or absence of available services.

## Discussion

This paper adds new insights to the limited evidence available on the specific effects that caring for a loved one at the end of life can have on bereavement experiences. These are based on the first hand experiences of family members, the second hand observations of health and social care professionals, or in many cases a combined perspective brought by bereaved professionals, which were articulated in ‘free text’ written responses to a self-completed survey. The data reported here demonstrates two core bereavement issues for family caregivers; the consequences of traumatic deathbed experiences on caregiver grief and feelings of guilt; and the ‘void’ effect caused by the withdrawal of professional support immediately after death, compounding feelings of loss for some recently bereaved family members. Support needs relating to these experiences are also identified with practical suggestions made for how such needs might be addressed.

One of the strongest themes to emerge in this data set concerned the impact of traumatic death experiences on family members’ abilities to grieve and adjust in the months and years that followed. As in Sanderson et al’s study reporting on death experiences and bereavement, the language of trauma was evident as respondents recounted their death bed experiences and appeared haunted by memories of the death [17]. It seems that some of our respondents also experienced an added critical dimension to their trauma and adjustment difficulties; living with guilt and regret. Whereas most participants in Sanderson et al’ s study engaged in positive self-reappraisal and were able to transform their trauma into more ‘bearable’ stories [17], these respondents not only perceived physical suffering in their loved ones, but also experienced a sense of powerlessness, exclusion and lack of understanding over what was happening. As a result, many also experienced feelings of lasting guilt and self-doubt over whether the right decisions were made and whether they could have done more. Although positive impacts of the LCP for families have been reported in other European countries [31, 32], these negative feelings were directly connected by some of our respondents to their first hand experiences of the implementation of the Pathway. Further, it seems that their doubts and sense of regret may have been compounded by media reactions, and the recent withdrawal of the LCP in the UK.

These kinds of guilt experiences are also evident in some of the literature reporting on the bereavement impacts of caregiving prior to the death, such as failure to provide EoLC at home, concerns and regrets over healthcare decisions that were made [15, 20, 22, 23], and increased, long term psychological morbidity amongst widowers who perceived un-relieved symptoms in their loved ones in the last three months of life [9, 10]. Moreover, the ‘good death’ experiences reported in this and other papers [25, 33, 34] and more positive stories of satisfaction and accomplishment reported by participants in other studies [8, 14–19], underline the importance of knowing that everything went as well as it could have done for the process of adjustment post-death.

Experiences of poor communication, resulting in limited understandings of the dying process and medical interventions amongst families, was a common factor in many of the troubled death and bereavement experiences reported here. Following this, several communication and support needs relating to death and dying were articulated. These included better information on the signs of death in order to facilitate awareness of and preparedness for death [12, 20, 25, 34], along with real time updates on what is happening during the dying process, and what relatives could be doing to help their loved ones at this time. Caregivers and health professionals also described the need for more effective communication and emotional support around EoLC planning and decisions, with detailed discussion at points where the focus of care shifts to prioritising EoLC goals, as evidenced elsewhere [30, 31]. Post death consultations with health professionals who were directly involved at the end of life were also recommended to alleviate some of the confusion, doubts and concerns of bereaved family members, the importance of which has been identified in other studies [35, 36].

Another grief experience specific to family carers was the reported “void” created by the withdrawal of services after death. Models of palliative care provision include extended support for the carers and families of patients during the period of illness, as well as intensive multi-professional support for the patient [3–5]. The ‘double loss’ experiences of carers has been reported elsewhere in terms of the ‘vacuum’ effect created by the loss of the caring role after death [16]. This ‘loss of relationships’ thus indicates a further loss experience for some carers, along with that of their caring role and the major loss of their loved one. It follows that many respondents would have liked continued contact between families and the healthcare organisations and professionals involved in the EoLC of their loved ones, as highlighted elsewhere [35–38]. A lack of information on, and access to, appropriate support was also identified in this and other studies [7]. Current recommendations for bereavement support in UK palliative care include targeted intervention based on formal risk assessment, coupled with universal information provision on grief responses and available support [2, 4]. These findings suggest unmet need in relation to both information provision and follow up support. Although most hospices in the UK offer bereavement support and many make contact with bereaved families at around six weeks post-death, these services have been noted as ‘idiosyncratic’ [38], while evidence guiding the timing, frequency, duration and nature of follow up support is lacking [36, 38].

### Clinical implications

Several implications for practice can be identified. First is the need for improved communication between health care professionals and families, in the period leading up to, and immediately following the patient’s death. As recommended elsewhere, family caregivers should be helped to recognise the signs of death in order to facilitate their preparedness for death [1, 12, 20, 25, 34] and be made aware when death appears imminent (1). Further, these findings suggest that preparedness for death should incorporate broader aspects of caring for the dying patient, such as physiological changes (e.g. airway secretions, the need or not for parenteral fluids), how to physically care for the dying body and what to do after death. In order to address caregiver experiences of marginalisation and lack of understanding relating to decisions made at the end of life, there also needs to be more effective engagement and discussion with families on end of life care planning and decisions, in particular when the focus of care shifts to prioritising end of life care goals [1, 29, 32]. As recommended in recent guidelines developed in Australia, it seems that many relatives would benefit from immediate post-death contact from a member of the care team, not only to offer condolences but also answer those potentially ‘nagging’ questions and concerns relating to the death [1].

These findings also indicate a need for better information provision on bereavement support prior to death and more effective follow up approaches, which in recent guidelines have been recommended to take place 3–6 weeks post death [1]. Such contact should inform, assess and connect family members with appropriate, available support (if required) [1, 35, 36], whilst also providing continuity and preventing carers from feeling “cut off” and experiencing the sense of multiple loss described above. As noted elsewhere, well conducted follow up visits also provide an opportunity to acknowledge the family member’s efforts during caregiving, thus also addressing feelings of guilt and supporting caregivers to find positive meaning in their experiences, as discussed above [36].

### Limitations and implications for research

The strength of this data set is the access which it gives to a wide range of perspectives on palliative care in the UK, and the breadth of detailed responses which were submitted on this and other topics of relevance to palliative care provision and research. These types of ‘free text’ survey responses can prove a rich, insightful source of data when analysed appropriately [39, 40], but there are also limitations. Given the survey aim and design it is likely that the responses represent more ‘extreme’ cases, particularly in relation to bad death experiences which may have left the respondent feeling compelled to speak out, and which contrast with some of the more mixed, even positive experiences reported in other qualitative studies [8, 14–19, 33, 34]. It is also more likely that respondents would have commented on service related problems or experiences than other aspects of their caregiving/ grief experiences which might be perceived as more inevitable (e.g., the decline of the patient). These more extreme and problematic experiences are nonetheless essential to capture, particularly in the context of complicated bereavement and grief issues. Because these responses were ‘fixed’ upon submission, it has also not been possible to unpack these experiences at an individual level or to pick up on points of interest as in interview based approaches, which are recommended for further in depth exploration of these issues. Future research which explores the characteristics and effectiveness of different communication and engagement approaches with families in the pre and post death periods is also recommended.

## Conclusion

This paper provides further insight into the relationship between caregiving and bereavement experiences. It identifies two core bereavement issues which are specific to people who have cared for loved ones at the end of life; the consequences of traumatic deathbed experiences on caregiver grief and the ‘void’ effect caused by the withdrawal of professional support immediately after death. Finding ways of improving communication around the time of death and identifying effective follow up approaches post death could help to address some of these issues.

## Abbreviations

EoLC: End of life care
LCP: Liverpool Care Pathway
PeolcPSP: Palliative and End of Life Care Priority Setting Partnership
